# Neurotoxins: Free Radical Mechanisms and Melatonin Protection

**DOI:** 10.2174/157015910792246236

**Published:** 2010-09

**Authors:** Russel J. Reiter, Lucien C. Manchester, Dun-Xian Tan

**Affiliations:** Department of Cellular and Structural Biology, University of Texas Health Science Center, San Antonio, Texas

**Keywords:** Aminolevulinic acid, cyanide, domoic acid, kainic acid, melatonin, metals, methamphetamine, polychlorinated biphenyls, rotenone, toluene, 6-hydroxydopamine.

## Abstract

Toxins that pass through the blood-brain barrier put neurons and glia in peril. The damage inflicted is usually a consequence of the ability of these toxic agents to induce free radical generation within cells but especially at the level of the mitochondria. The elevated production of oxygen and nitrogen-based radicals and related non-radical products leads to the oxidation of essential macromolecules including lipids, proteins and DNA. The resultant damage is referred to as oxidative and nitrosative stress and, when the molecular destruction is sufficiently severe, it causes apoptosis or necrosis of neurons and glia. Loss of brain cells compromises the functions of the central nervous system expressed as motor, sensory and cognitive deficits and psychological alterations. In this survey we summarize the publications related to the following neurotoxins and the protective actions of melatonin: aminolevulinic acid, cyanide, domoic acid, kainic acid, metals, methamphetamine, polychlorinated biphenyls, rotenone, toluene and 6-hydroxydopamine. Given the potent direct free radical scavenging activities of melatonin and its metabolites, their ability to indirectly stimulate antioxidative enzymes and their efficacy in reducing electron leakage from mitochondria, it would be expected that these molecules would protect the brain from oxidative and nitrosative molecular mutilation. The studies summarized in this review indicate that this is indeed the case, an action that is obviously assisted by the fact that melatonin readily crosses the blood brain barrier.

## INTRODUCTION

The brain, including the spinal cord, is a very highly metabolically active organ which, even at rest, utilizes an estimated 20% of the total oxygen taken up by the lungs. This percentage increases substantially when the brain is active. Depriving the brain of its rich oxygen supply even for short intervals of a couple of minutes often has severe and irreversible detrimental morphological and physiological consequences for both neurons and glia. Clearly then, the brain more so than any other tissue, is reliant on an uninterrupted oxygen-rich blood supply for its survival.

This high utilization of oxygen, however, comes at a heavy biological price. As important as oxygen is for the survival of neurons and glia, it also indirectly contributes to their destruction and death over time. The reason for this is that a small percentage (an estimated 1-4%) of the oxygen that enters cells is metabolized to derivatives that gradually erode and destroy essential molecules [30, 86, 148]. These destructive derivatives of oxygen are often referred to as free radicals (although some are not radicals per se) or reactive oxygen species (ROS). Some of the most noteworthy destructive oxygen metabolites include the superoxide anion radical (O_2_•-), hydrogen peroxide (H_2_O_2_) and the hydroxyl radical (•OH); this latter agent is especially toxic to cells and indiscriminately harms or destroys any molecule in the vicinity of where the radical is generated [178]. O_2_•-, albeit not especially reactive, combines with nitric oxide (NO•), to produce the peroxynitrite anion (ONOO^-^), a non-radical product which is generally believed equivalent to the •OH in terms of its ability to oxidize and destroy bystander molecules. NO• and ONOO^-^ are often referred to as reactive nitrogen species (RNS).

Cells must be equipped with means to quell the molecular damage meted out by ROS and RNS; if they have inefficient systems by which to protect themselves, the life of cells becomes dire. When intracellular molecules are mutilated by ROS/RNS, the cells become metabolically compromised eventually leading to cell death. When cells die and they are not replaced, such as occurs in most areas of the brain, the tissue falters and eventually organismal death is an unavoidable consequence. 

To combat free radical (ROS/RNS) destruction, all life forms have evolved an array of means to either quench radical generation, to convert radicals to metabolically non-destructive molecules, or to neutralize (scavenge) them immediately after they are formed. This complex protective system which is designed to prevent radical-mediated organ malfunction, disease processes and aging is referred to as the antioxidative defensive system.

Remarkably, the brain is not especially effective at protecting itself from ROS/RNS. The reasons for this are several-fold. Most importantly, as already mentioned, the CNS produces a disproportionately high number of ROS/RNS because of its use of seemingly more than its share of oxygen, thereby generating more than the usual number of destructive ROS/RNS. The brain, seemingly unexpectedly, is relatively deficient in the enzymes that metabolize a number of the oxygen-based reactants to innocuous species. The CNS is highly enriched with polyunsaturated fatty acids which, more so than many other molecules, are readily oxidized by toxic oxygen derivatives. Some areas of the brain contain large amounts of vitamin C (ascorbic acid); while vitamin C is an antioxidant capable of scavenging free radicals, in the presence of free iron, it functions as a potent pro-oxidant. This often occurs when there is hemorrhage in the CNS where escaped erythrocytes degenerate releasing hemoglobin which then deteriorates leaving in its wake iron molecules. The blood-brain barrier, which is designed to protect the brain from toxins by limiting their diffusion into neurons and glia, also curtails the uptake of some protective agents, e.g., the antioxidant vitamin E, into the brain. This combination of features, or deficiencies, makes the brain highly susceptible to molecular destruction by ROS/RNS.

The current review is concerned with molecules, drugs or other agents which enter the brain and directly or indirectly generate ROS/RNS which destroy essential molecules leading to neuronal and glial loss and malfunction. Moreover, the survey reviews the ability of the endogenously-produced antioxidant, melatonin, to battle against free radical damage and to protect the brain from molecular mutilation which would inevitably lead to motor and sensory disability, psychological disturbance and/or cognitive decline.

## MELATONIN AS AN ANTIOXIDANT

The processes by which melatonin quells molecular damage resulting from oxygen derivatives are only briefly enumerated here since there are numerous reviews on this subject [4, 5, 90, 93, 189-191, 194, 196, 197, 244, 245]. The results summarized in these surveys should assure the reader of the high efficacy of melatonin and its derivatives in screening all cells, including those in the CNS, from molecular havoc.

N-acetyl-5-methoxytryptamine (melatonin), was discovered as a potent antioxidant in 1993 [236]. Since then, melatonin’s ability to protect all cells and organs from oxidative/nitrosative damage has been confirmed in more than a thousand publications [17, 79, 128, 178, 197]. The indoleamine melatonin is produced in all animals from unicells to humans [71, 91, 92, 141, 248, 259] and also in plants [14, 60, 94, 142, 159, 162, 172, 195, 240, 241]. Whereas melatonin has many other functions in organisms, i.e., helping to synchronize circadian rhythms [6, 12, 102, 106, 112, 175], sleep promotion [12, 69, 105, 261, 274], immune stimulation [40, 82, 127, 146, 209, 210], blood pressure regulation [35, 65, 200, 218, 225, 226,], seasonal reproductive regulation [44, 134, 187, 188, 201, 235], oncostatic [26-28, 51, 97, 110, 120, 199, 214], antidepressive [113, 123, 140, 157, 192], etc., it seems to be unparalleled as an antioxidant. The ability of melatonin to protect cells from free radical injury extends from plants to humans [13, 41, 118, 179, 240, 241].

Melatonin’s functional repertoire in terms of limiting molecular destruction by both oxygen and nitrogen-based radicals and associated metabolites is highly diverse. This indoleamine functions at all levels to aid in the ability of organisms to resist the onslaught of damage normally inflicted by radicals and radical-related products. Thus, melatonin reduces free radical generation at the mitochondrial level in a process generally referred to as radical avoidance [88, 135, 147, 171], it stimulates antioxidative enzymes that convert highly toxic species to innocuous products [11, 18, 43, 87, 168, 169, 208, 250], it promotes the synthesis of another antioxidant, glutathione [252, 263], and it inhibits at least one enzyme, nitric oxide synthase, that normally produces free radicals, in this case NO• [20, 26, 129, 181].

Not only does melatonin carry out these functions to help cells avoid molecular damage, but it also directly neutralizes free radicals. In addition to melatonin itself, several of the metabolites that are formed when melatonin scavenges radicals also participate in antioxidative defense by incapacitating toxic species [58, 63, 76, 89, 93, 143, 144, 149, 150, 176, 232, 244, 275, 276] and by modulating the activities of enzymes that maintain the redox balance within cells [11, 168, 169, 180, 213, 260, 270]. These functionally critical metabolites of melatonin include cyclic 3-hydroxymelatonin [137, 242], N1-acetyl-N2-formyl-5-methoxykynuramine [32, 114, 224, 241, 243, 245], N1-acetyl-5-methoxykynuramine [81, 89, 124, 206, 217], and perhaps others. Some of the antioxidative actions of melatonin, e.g., stimulation of antioxidative enzymes [18, 61, 168, 169, 202, 203, 208, 250] are likely receptor-mediated while others are receptor-independent, e.g., direct radical scavenging [108, 109, 171, 202, 236, 273].

*Via *these collective mechanisms, melatonin appears to be an uncommonly efficient protector of molecules, subcellular organelles, cells, tissues and organisms from both harm as well as from death *via *apoptosis or necrosis due to a wide variety of toxic agents [66, 70, 119, 161, 167, 204, 223, 272]. The subsequent paragraphs describe the results of studies where melatonin was used to protect neural tissue from toxic agents that damage the brain because of their ability to elevate the production of ROS and/or RNS.

## METHAMPHETAMINE

Methamphetamine (METH) is a drug of abuse that produces long-lasting neurotoxic effects on the CNS. Neurochemically, this drug has potent inhibitory effects on tyrosine hydroxylase (TH) [116] and tryptophan hydroxylase (TPH) [100, 215]. These changes are associated with a drop in striatal dopamine (DA) [100, 233] and serotonin (5-HT) [16, 219] levels as well as a reduction in the transporters for both of these neurotransmitters [121, 207]. Free radicals are suspected of being involved in these responses since transgenic mice that express human copper/zinc superoxide oxide dismutase (CuZn SOD) exhibit an attenuated response to METH in terms of the reductions in neural DA and 5-HT concentrations [34, 55]. Indeed, O_2_•- generation is considered a major culprit in the toxicity of METH [99]. 

Considering the likely involvement of ROS in the toxicity of METH to neuronal monoaminergic systems, experiments designed to attenuate the negative effects of METH using melatonin have been conducted. Unexpectedly, the first study performed claimed that melatonin (25 mg/kg), co-administered with METH (15 mg//kg), actually exacerbated the neural deficits that normally occur after METH administration [75]. All subsequent experiments, however, have yielded diametrically opposite outcomes, i.e., melatonin attenuated METH damage to the CNS [9, 98, 101, 111, 117, 234, 249, 264]. The doses of the respective drugs used by Gibb and colleagues [75] as well as the endpoints measured were similar as those employed by numerous other investigators, so an explanation as to why their findings so markedly differ is not readily apparent. The overwhelming consensus is that melatonin reduces or overcomes the neural toxicity of METH.

In the first study that revealed contrary findings to those reported by Gibb *et al.* [75], Hirata *et al.* [98] used melatonin doses ranging from 5-80 mg/kg that were intraperitoneally injected 30 minutes in advance of METH administration (4 x 5 mg/kg) given at 2-hour intervals. Melatonin reduced the inhibitory effects of METH on both the DA and 5-HT transporters in the striatum and nucleus accumbens of mice; these findings were documented using both biochemical and autoradiography methods. The obvious conclusion was that melatonin is protective against METH toxicity, at least in regard to the parameters measured and the brain areas examined. 

The following three reports focused specifically on the ability of melatonin to modify the responses of neural DA metabolism to METH [9, 101, 104]. Using a variety of endpoints, it was shown that under all experimental situations tested melatonin reversed the effects of METH. These workers speculated that melatonin’s beneficial actions in METH-treated rodents may stem from its ability to reduce the hyperthermic action of the drug of abuse [9, 104]. The ability of melatonin to modify the body temperature-altering effect of METH is expected considering that melatonin by itself also reduces body temperature. 

In the study of Imam *et al.* [101], ONOO^-^ was examined as a possible contributor to METH toxicity by measuring neural 3-nitrotyrosine (3-NT) formation. A single injection of METH produced a significant rise in 3-NT concentrations in the striatum signifying the involvement of ONOO^- ^in the destructive effects of this drug of abuse. Melatonin completely prevented the rise in 3-NT levels as well as reversing the depletion of striatal DA. The findings are consistent with the ONOO^-^, an RNS and a strong oxidizing agent, being a participant in the damage to the brain inflicted by METH. Since melatonin has been shown to scavenge the highly toxic ONOO^-^ [25, 160, 278], it seems likely that the protective actions of melatonin against the neural damage inflicted by METH may involve its direct detoxification of the ONOO^-^.

Since the immature brain is relatively deficient in an antioxidative defense system, Kaewsuk and co-workers [111] anticipated that treating neonatal rats with the antioxidant melatonin would prevent at least some of the toxicity of METH in the newborn animals. METH (5-10 mg/kg) was given to rats on postnatal days 4 through 10. Each subcutaneous METH injection was preceded by a dose of 2 mg/kg melatonin. The drug of abuse reduced TH activity in the dorsal striatum, prefrontal cortex, nucleus accumbens and substantia nigra, while also lowering synaptophysin levels in the striatum and prefrontal cortex and growth-associated protein-43 in the nucleus accumbens. Each of these changes was significantly attenuated in the neonatal mice that were given, in addition to METH, melatonin. The protective effects of melatonin in this study were presumed to be related to the direct scavenging and indirect antioxidant activity of melatonin. 

To further test the role of melatonin’s antioxidative potential in mitigating the destructive effects of METH, *in vitro* studies using both cultured neuroblastoma and microglial cells were performed. Wisessmith *et al.* [264] and Suwanjang and colleagues [234] added METH to the medium in which human neuroblastoma cells (SH-SY5Y) were growing. The drug significantly reduced cell viability and TH activity while upregulating Bax, Bcl-2, cleaved caspase-3 protein and calpain expression as well as reducing the endogenous calpain inhibitor, calpastatin. Each of these changes was abolished when METH-treated SH-SY5Y cells were also incubated with melatonin. The results of these studies document the induction of death signaling proteins by METH and further show that melatonin can reverse the effects of METH on these signaling pathways thereby preserving neuronal viability.

While ROS and RNS are accepted as contributors to neuronal death following METH administration, the effects of this drug on neuroinflammation have been less thoroughly tested. To examine this, aggressively proliferating immortalized rat microglial (HAPI) cells were used [249]. As with neurons, the addition of METH reduced microglial cell viability in a concentration and time-dependent manner while upregulating the expression of inflammatory cytokines, interleukin 1β (IL-1β), interleukin-6 (IL-6) and tumor necrosis factor alpha (TNF-α). Moreover, ROS and RNS generation was exaggerated as a result of METH administration; these were monitored using fluorescent probes. Melatonin abolished all aspects of METH toxicity in this study. Clearly, the findings show that inflammatory cytokines as well as ROS and RNS may be involved in METH toxicity in the brain. That melatonin reversed these effects is consistent with its antioxidant and anti-inflammatory properties [24, 197, 209, 254].

Notwithstanding the one outlying report which claimed that melatonin actually enhanced the toxicity of METH [75], all subsequent investigations proved otherwise. Using multiple endpoints, the published reports collectively support the idea that the antioxidant and anti-inflammatory actions of melatonin contribute to the ability of this indoleamine to mitigate the toxic neural effects of METH.

## AMINOLEVULINIC ACID

Acute intermittent porphyria (AIP) is associated with functional defects in both the central and peripheral nervous systems [271]. The pathophysiology that develops in this case is generally accepted as being secondary to the generation of ROS [59, 95]. AIP is characterized by elevated levels of δ-aminolevulinic acid (ALA), the agent likely responsible for the generation of oxygen-centered reactants in neural tissue [96]. Interestingly, patients with AIP also reportedly have depressed nocturnal levels of melatonin [184].

Given the role of oxygen-based derivatives in the toxicity of ALA, Princ and co-workers [182] tested the efficacy of pharmacological concentrations of melatonin in reducing lipid peroxidation in rat cerebellar tissues treated with ALA. ALA-mediated accumulation of lipid hydroperoxides was reduced, in a dose-dependent manner, by melatonin. These findings confirmed the likely involvement of free radicals in ALA-toxicity as well as showing that melatonin could potentially be useful in overcoming the damaging effects of ALA. Whereas melatonin treatment required pharmacological levels of melatonin, likewise the concentrations of ALA used to enhance the oxidation of lipids was also much higher than would ever be achieved *in vivo*. Thus, physiological concentrations of melatonin, or any oxidant, could not prevent the toxicity of such high levels of ALA.

These studies were extended by Carniero and Reiter [37] who showed that melatonin had a similar protective action against ALA-induced lipid peroxidation in the hippocampus and cerebral cortex just as it had in the cerebellum. In this study malondialdehyde and 4-hydroxyalkenals were measured as oxidative stress endpoints. This group also showed that *in vivo* as well as, the oxidation of cerebellar and hippocampal lipids resulting from ALA administration was attenuated when melatonin was acutely administered before ALA was injected. 

*In vivo* studies were also carried out by Princ and co-workers [183] and they also reported melatonin’s ability to overcome ALA-induced neural lipid peroxidation. Moreover, melatonin treatment was found to preserve the activities of both porphobilinogen deaminase and δ-aminolevulinate dehydratase in the cerebral cortex. While the authors surmised that melatonin’s protective actions in part related to its ability to directly scavenge toxic reactants, they also felt that the indoleamine’s ability to protect against free radical damage was a result of its ability to stimulate antioxidative enzymes. Whereas they did not measure these activities, they based their supposition on earlier studies that showed that melatonin is certainly capable of stimulating antioxidant enzyme activity [18, 168, 169].

## 6-HYDROXYDOPAMINE

The injection of the catecholaminergic neurotoxin, 6-hydroxydopamine, directly into the substantia nigra of the midbrain leads to degeneration of the dopamine (DA)-producing parikarya and subsequently the degeneration of the nigra-striatal pathway causing Parkinson-like signs in animals [54, 62]. Since the injection of the toxin is typically done unilaterally, the lesions develop only on one side with the resulting model being referred to as hemiparkinsonism [52, 228]. The neurodegenerative lesion is believed to involve oxidative damage, mitochondrial malfunction, release of excitatory amino acids and a rise in intracytoplasmic free calcium [47, 163]. The neurons eventually die *via *apoptosis [31]. Given the involvement of oxidative damage and other downstream actions of 6-hydroxydopamine, it was anticipated that melatonin may ameliorate some of the effects of this drug. Both *in vitro* and *in vivo* studies have been carried out to test this possibility. 

Mayo and co-workers [152] reported that the incubation of PC-12 neurons with 25 or 50 µM 6-hydroxydopamine killed a large percentage of naïve PC-12 cells. Melatonin, added at a concentration of either 10^-7^ to 10^-9^ M highly significantly reduced cell death mediated by the neurotoxin. Likewise, melatonin lowered DNA fragmentation resulting from 6-hydroxydopamine incubation in the PC-12 neurons. Besides functioning as a direct free radical scavenger in this study, melatonin also probably altered the PC-12 cell response by adjusting the activities of two key antioxidative enzymes, MnSOD (manganese superoxide dismutase) and Cu/Zn SOD. Morphologically, melatonin preserved the ultrastructural morphology of PC-12 neurons cultured with 6-hydroxydopamine further confirming the protective actions of melatonin against the neurotoxin [151]. 

The *in vitro* protective actions of melatonin against 6-hydroxydopamine toxicity was also documented in SK-N-SH cells [46]. In these cells as well, melatonin preserved cell viability as revealed by the MTT (3- (4, 5-dimethylthiazol-2-yl) -2, 5-diphenyl-tetrazolium bromide) assay. Moreover, the ability of melatonin to reduce neuronal cell death involved inhibition of the c-Jun-N terminal kinase signaling cascade. On the basis of these findings, the authors urged consideration of the use of melatonin in clinical trials of free radical-related neurodegenerative conditions.

In a series of two brief reports, a group of Korean scientists [107, 115] were the first to examine melatonin’s ability to reduce the toxicity of 6-hydroxydopamine *in vivo*. In these studies, the unilateral Parkinsonism model was used. The neurotoxin caused rises in the level of malondialdehyde (142%) and reductions in TH (28%), DA (32%) and the dopamine metabolite, dihydroxyphenylacetic acid (50%) on the side of the lesion two weeks after the toxin had been stereotoxically injected. In the behavioral aspects of the study, melatonin also attenuated rotational asymmetry induced by the injection of the dopamine receptor agonist, apomorphine. Melatonin also enhanced survival of the dopaminergic cell bodies in the substantia nigra and the tyrosine-immunoreactive nerve terminals in the dorsolateral striatum as revealed by immunocytochemistry.

Similar observations were reported by Agular and co-workers [7]. In this case the 6-hydroxydopamine was injected into the striatum; the toxin caused significant decreases in DA, DOPAC (3, 4-dihydroxyphenyl acetic acid) and HVA (homovanillic acid). Melatonin, given in doses ranging from 2-25 mg/kg (given intraperitoneally for 7 days after 6-hydroxydopamine injection) caused a partial reversal (roughly 50%) of the losses in these neurotransmitters. As did Joo *et al.* [107] and Kim and colleagues [115], Agular *et al.* [7] also noted that melatonin prevented the abnormal rotational behavior of the rats following an apomorphine injection. Finally, melatonin produced an upregulation of D1 receptor with a reduction in the Kd value. 

It was also of interest to test the ability of melatonin to reduce the deficits in mitochondrial oxidative phosphorylation that are a result of unilateral 6-hydroxydopamine injections into the substantia nigra [56]. Using a novel BN-PAGE histochemical procedure, it was shown that mitochondrial complex I activity was significantly compromised in the 6-hydroxydopamine-damaged neurons in the substantia nigra of rats. In rats implanted with subcutaneous mini-pumps that delivered a constant 50 ± 7.5 µg melatonin per hour, the suppressed complex I activity was completely prevented. 6-hydroxydopamine also caused a non-significant reduction in complex IV activity with this drop also being reversed by the continuously-available melatonin. 

Challenging the 6-hydroxydopamine-damaged rats with apomorphine caused the characteristic rotational behavior. There was a strong correlation in terms of the reduction in complex I activity and the severity of the rotational response. As in previously-reported studies, melatonin prevented the abnormal rotation, a response again correlated with the preservation of mitochondrial complex I activity.

That physiological levels of melatonin may be relevant to reducing 6-hydroxydopamine toxicity has also been investigated [221]. In this study, melatonin was administered in the drinking fluid at a concentration of either 0.4 or 4.0 µg/ml). The authors had calculated that these concentrations of melatonin, when consumed by hemi-parkinson rats, would cause physiological levels of melatonin normally present in the blood and cerebrospinal fluid, respectively (cerebrospinal fluid, from the third ventricle, contains much higher concentrations than those measured in nighttime collected blood). The highest dose of melatonin significantly attenuated rotational behavior compared to that observed in rats given vehicle or the lower melatonin dose. When the brains of these animals were examined, those from rats given 4.0 µg/ml drinking fluid exhibited normal TH immunoreactivity in the striatally-lesioned rats; in contrast, rats not given melatonin were essentially devoid of TH activity at the lesioned site. In comparison, when mRNA levels for TH were estimated there were no differences among the groups suggesting, according to the authors of the report [221], a spontaneous recovery of this transcript.

Two particularly novel publications have appeared in the last several years related to the issue of melatonin in relation to experimental Parkinsonism. In the first of these, C17.2 neural stem cells (NSCs) were transplanted into the substantia nigra of 6-hydroxydopamine-lesioned rats with or without melatonin co-administration [220]. In these animals, behavioral studies documented that NSC transplantation, melatonin administration, or a combination of both treatments significantly reduced abnormal rotational behavior. Moreover, each of the procedures reduced the loss of TH immunoreactivity in the striatum resulting from 6-hydroxydopamine administration. The findings reported in this beautifully-illustrated paper document that combining NSC transplantation with melatonin treatment may improve outcome and, therefore, it could be a viable approach to use in the treatment of Parkinson disease.

L-DOPA (L-3, 4-dihydroxyphenylalanine) administration is used to restore DA levels in the brain of individuals diagnosed with Parkinson disease. The rise in neuronal DA causes the production of 6-hydroxydopamine [139], a molecule, as summarized above, that has significant neural toxicity. It is presumed that the neural toxin is a consequence of the high production of ROS at the site of the lesion in the substantia nigra. Given the presumed involvement of oxygen-based radicals *in vivo* 6-hydroxydopamine production, it was surmised that melatonin would likely inhibit the production of the neurotoxin. Using a ferrous-ascorbate-dopamine (FAD) hydroxyl radical generating system, it was found that melatonin prevented 6-hydroxydopamine formation from DA *in vitro* [29]. When FAD was infused into the medial forebrain bundle in the diencephalon of mice, a significant depletion of DA was recorded in the striatum. Treatment of mice with 30 mg/kg melatonin 30 minutes in advance of L-DOPA administration reduced 6-hydroxydopamine production in the brain and protected against DA depletion in the striatum.

Collectively, the studies summarized in this section of the review convincingly document the utility of melatonin in nullifying the toxicity of the neurotoxin, 6-hydroxydopamine, and in preventing DA loss in neuronal cell bodies in the pars compacta of the midbrain and in the nerve terminals in the striatum. This being the case, it seems reasonable to suggest the use of melatonin as an experimental treatment of Parkinson disease. Its use for this purpose is emphasized by the essential lack of toxicity of this indoleamine and the ability to administer it *via *essentially any route. 

Finally, given that L-DOPA is commonly used to treat Parkinson patients and the fact that it, while restoring DA levels, also induces 6-hydroxydopamine production in a process that involves free radicals, melatonin should be tested in combination with L-DOPA to determine if it would improve the longterm outcome of these patients. Additionally, when NSCs are transplanted into the brain, the use of melatonin may prove worthwhile. 

## ROTENONE

Rotenone, a pesticide, reproduces key behavioral symptoms and neuropathological features of Parkinson disease [23]. The changes include apomorphine-mediated rotational motor problems, degenerative changes in the nigrostriatal dopaminergic pathway, elevated oxidative stress and changes in the brain reminiscent of Lewy bodies; the intraneuronal inclusions that form are polyubiquitin and α-synuclein-positive [21, 22, 36]. Also, rotenone causes mitochondrial complex I inhibition leading to a selective depletion of DA from neurons of the substantia nigra [21] which obviously contributes to its toxicity [222].

The first test of melatonin’s ability to inhibit rotenone toxicity used the fruit fly *Drosophila melanogaster*, as the experimental model [53]. This species is not an uncommon model in which to investigate drugs which influence neurodegenerative diseases. Treatment of flies with rotenone exhibited characteristic locomotor impairments and examination of the brain clusters revealed a dramatic and selective loss of immunocytochemically-identified DA. When L-DOPA was incorporated into the diet of the flies, the behavioral deficits were alleviated but brain DA was not restored; this is similar to L-DOPA administration in the human. Adding melatonin to the diet substantially alleviated both the behavioral abnormalities and, importantly, also prevented loss of DA neurons. The authors suggested the consideration of melatonin as a treatment for Parkinsonism in humans. 

The intranigral infusion of rotenone caused the depletion of reduced glutathione (GSH) as well as changes in SOD and catalase in rats [216]. With the aid of a sensitive HPLC method, it was also found that rotenone enhanced •OH generation in submitochondrial particles. Especially the measured free radical generation, but also the changes in GSH and antioxidative enzymes induced by rotenone were prevented when pesticide treatment was coupled with melatonin.

When rotenone was infused subcutaneously for 14 days into rats, it produced Parkinson-like signs including lowered locomotor activity (hypokinesis), marked reduction in TH immunocytochemistry in the substantia nigra and striatum, (α-synuclein accumulation, downregulation of the dopamine transporter and upregulation of the D2 dopamine receptor [133]. These workers also examined the locus coeruleus and noted that rotenone depleted these neurons of norepinephrine. Melatonin (10 mg/kg/day) administration prevented the changes observed in the nigrostriatal system and α-synuclein aggregation; melatonin did not, however, alleviate the obvious hypokinesis seen in the rotenone-treated rats. Finally, melatonin also reduced the downregulation of the dopamine transporter and the upregulation of the D2 receptor. On the basis of the outcome of this thorough series of investigations, it was concluded that rotenone toxicity is at least partially related to free radical damage and that melatonin may be useful to counteract some of the degenerative changes associated with Parkinsonism [133].

There is one study in which the findings run contrary to those reported in the previous paragraphs. Thus, when melatonin (total of 20 mg/kg daily; 10 mg/kg X 2 daily) was administered to rotenone-treated male Lewis rats (a strain not used in other reports that showed melatonin protected against rotenone), it failed to reverse the depletion of DA and its metabolites (DOPAC and HVA) in the striatum induced by the pesticide [246]. Indeed, melatonin actually further lowered the striatal depletion of these constituents caused by rotenone. Likewise, the authors reported that melatonin further diminished striatal and nigral TH immunoreactivities. Why the findings reported differ so widely from a number of other studies which revealed that melatonin reverses the suppressive effect of rotenone and other neurotoxins on neural DA levels was not readily apparent to the authors of the report. They did, however, make one curious observation that could have accounted for the unusual responses that they observed in the rotenone-injected rats. Thus, in control animals given melatonin only, striatal DA levels were significantly elevated over those in all other groups; whether this was also true in the substantia nigra was not reported. According to the authors, a rise in striatal DA may have potentiated the effects of rotenone-induced complex I inhibition and thereby elevated the degree of oxidative stress. Since Tapais *et al.* [246] did not include any measures of free radical damage in their studies, they proposed that this should be a goal of further mechanistic studies.

In summary, the interaction of melatonin and rotenone in terms of DA metabolism, as assessed at the level of the striatum and substantia nigra, requires further investigation. While the majority of studies found melatonin to be protective against the neurotoxicity of rotenone, the final report discussed in the previous paragraph noted otherwise. Moreover, there is no ready explanation for the different outcomes of the summarized publications. This issue requires resolution with additional experimentation since the findings could, theoretically at least, have implications for other drugs that also disturb the dopaminergic system.

Another frequently used drug to induce Parkinson-like signs in experimental animals is 1-methyl-4-phenyl-1, 2, 3, 6-tetrahydropyridine (MPTP). This subject is not covered in the current review since there are a variety of recent publications and reviews on this subject [3, 45, 48, 153, 209]. Likewise, with the exception of the findings related to aluminum administration, the ability of melatonin to resist the development of Alzheimer disease signs following drug administration is not discussed in this review since Alzheimer disease is considered in another chapter in this volume and there is extensive literature published on this subject [164, 170, 209].

## METALS

The toxicity of metals is often explained on the basis of their ability to cause the generation of oxygen and nitrogen-based reactants [132, 253]. Iron (Fe), chromium (Cr), aluminum (Al), copper (Cu), vanadium (V) and cobalt (Co) undergo redox cycling reactions thereby permitting massive free radical production. Other metals including mercury (Hg), cadmium (Cd) and nickel (Ni) have the ability to deplete the important endogenous antioxidant glutathione, and also to bind to sulfhydryl groups on proteins. Several of these metals participate in the Fenton reaction which produces the highly reactive •OH. In general, antioxidants (both enzymatic and non-enzymatic) combat the deleterious actions of metals in the central nervous system, provided they are capable of crossing the blood-brain barrier.

Al has been implicated in the etiology of Alzheimer disease, although its contribution to this debilitating neurodegenerative condition still remains unresolved [122, 155]. Given the potential association of Al with Alzheimer dementia, molecules or antioxidants that limit its toxicity in the central nervous system are of obvious interest.

That melatonin may be capable of reducing oxidative damage to the central nervous system in Al-treated rodents has been examined in a number of publications. Accordingly, Esparza *et al.* [68] intraperitoneally injected Al into male rats with or without concurrent melatonin administration. At necropsy, the cerebral cortex, hippocampus and cerebellum were examined for their Al content; Al was in especially high levels in the cerebellum in the animals that received the metal. While melatonin did not change Al uptake by the brain, the results of this study clearly documented that Al is a pro-oxidant in the brain while melatonin reduced the molecular damage resulting from Al toxicity. The endpoints in this initial study were indirect measures of oxidative stress and included activities of glutathione-metabolizing enzymes and the neural levels of lipid peroxidation. The same group showed in two subsequent reports that the mRNAs for antioxidant enzymes were elevated in the cortex and cerebellum [67] and the hippocampus [80] of Al-treated rats that had also received melatonin. The daily (5 days a week) doses of Al and melatonin in this 11-week study were 7 mg/kg and 10 mg/kg, respectively. Since melatonin did not influence the neural uptake of Al, it was presumed that its protective actions related to its free radical scavenging activity and its ability to stimulate antioxidative enzymes. Despite the benefits of melatonin in terms of promoting antioxidative enzyme mRNA expression and activity levels in Al-treated animals [72-74], the indoleamine did not change the behavioral outcomes of transgenic mice (Tg2576; they express elevated levels of amyloid-β-precursor protein) in the open field and Morris water maze tests [74].

While this series of studies, all from the same laboratory, related to the apparent protective actions of melatonin against neural damage induced by Al administration to rodents suggests some benefits of administration of the indoleamine, there are only sparse data documenting that melatonin actually reduced the degree of neural oxidative damage that may have resulted from Al toxicity. This question should be addressed in any subsequent investigations related to the interactions of Al and melatonin in the central nervous system. In a related study in a non-rodent species, melatonin reduced the neural toxicity of chronic Al (as aluminum chloride; 20 mg/l *via *the drinking fluid) administration to rabbits [2]. Neural malondialdehyde and 6-hydroxyalkenal levels, indicative of lipid peroxidation, were elevated as a result of Al consumption and were reduced in the melatonin-treated rabbits. Neuropathological examination of the brain also suggested a protective action of melatonin against Al neurotoxicity. 

Co, a positively charged transition metal, has been found to be elevated in the postmortem brain of Alzheimer’s disease patients [247] with a particular high concentration in the nucleus of neurons [262]. When SHSY5Y neuroblastoma cells were incubated in serum-free medium containing 300 µM CoCl_2_, the metal induced cytotoxicity and cell death as well as inducing the release of amyloid-β [165]. When 1 µM melatonin was also added to the incubation medium, the deleterious actions were reversed. Whether melatonin would protect against the neural toxicity of Co *in vivo* has not been tested. 

Fe is a transition metal that is commonly implicated as contributing to oxidative damage due to its participation in the Fenton reaction which results in the generation of the highly reactive •OH. Fortunately, most Fe *in vivo* is bound and, therefore, cannot participate in the Fenton reaction. Although there are limited *in vivo* studies on the efficacy of melatonin in protecting against free Fe toxicity, *in vitro* it readily reduces molecular damage in cells resulting from their exposure to Fenton reagents [136]. In this capacity, melatonin was more effective in protecting against DNA damage than xanthurenic acid, resveratrol (3, 4’, 5-trihydrotrans-stilbene), EGCG [(-)-epigallocatechin-3-gallate], vitamin C or alpha-lipoic acid. A hepatic metabolite, 6-hydroxymelatonin, also was shown to reduce Fe toxicity after the infusion of ferrous citrate directly into the hippocampus of rats; the metabolite was, however, less potent than melatonin itself in reducing hippocampal lipid peroxidation and neural damage [138]. 

The toxicity of the heavy metal, lead (Pb), is well documented [1]. The nervous system, among many organs, is negatively influenced by Pb ingestion [211, 257, 277]. The negative neural consequences of this heavy metal are generally accepted as involving disturbances in the mitochondria which causes the generation of excessive numbers of free radicals [103, 258]. Armed with this information, El-Sokkary and colleagues [64] treated rats with either Pb or Pb plus melatonin to determine whether the indoleamine would modify the degree of oxidative damage in the brain of rats. As anticipated, rats injected subcutaneously with lead acetate for 21 days had high levels of malondialdehyde and 4-hydroxyalkenals in the brain. Additionally, reduced glutathione levels and diminished SOD activity were also apparent. Giving melatonin injections in advance of lead acetate essentially reversed all the oxidative damage that accompanied the neural toxicity of Pb.

Hg is frequently ingested in the diet and is becoming an increasingly common environmental contaminant. When rats were treated with mercuric chloride (4 mg/kg), it induced a variety of changes in the cerebral cortex, cerebellum and brain stem (medulla) [186]. The changes were estimated to be most severe in the cerebral cortex. Co-administration of melatonin (5 mg/kg) attenuated the neural changes. While these findings suggest melatonin may be protective of the brain against Hg toxicity, a solid conclusion to this effect should not be based on the results of this single study. 

Using absorptive cathode stripping voltammetry, two reports [124, 131] tested whether melatonin binds metals thereby potentially altering how they impact neural tissue. The studies revealed that melatonin does interact with Cu, Cd, Fe^3+^, Pb, Zn and Al. Due to these chelation reactions, melatonin could retard the Fenton reaction and thereby prevent •OH generation. Alternatively, attenuated endogenous melatonin levels could predispose to the buildup of toxic metals which would likely contribute to molecular damage in the brain and elsewhere. 

## TOLUENE

Toluene vapor inhalation causes adverse effects in the central nervous system with associated behavioral abnormalities [15, 125]. When rats were chronically exposed to toluene, elevated levels of lipid peroxidation were also measured in the cerebral cortex, hippocampus and cerebellum; the administration of melatonin, however, counteracted the effects of toluene and returned lipid oxidation to essentially control levels [19]. Subsequently, Pascual and associates [173] conducted a thorough investigation related to the potential protective actions of melatonin in protecting dendrite development in young rats that inhaled toluene vapors (5,000-6,000 ppm for 10 minutes daily for 12 days). This exposure strategy was designed to simulate the situation of toluene abuse in humans where individuals are exposed to high concentrations of vapor for repeated short intervals. When the brains were collected and analyzed 7 days after the last toluene inhalation, there was a marked suppression of dendritic outgrowth in layer II and III pyramidal neurons in the cerebral cortex; also of the dendrites that did grow the number of branches was significantly attenuated. These findings documented that subchronic toluene inhalation in young rats had a dramatic effect on basal dendrite development and branching in the frontal, parietal and occipital pyramidal neurons. When melatonin was given at a dose of 5 mg/kg, it reversed the neural damage. Thus, melatonin was clearly protective, likely *via *its antioxidative mechanisms, against toluene toxicity.

## POLYCHLORINATED BIPHENYLS

The neurotoxic effects of polychlorinated biphenyls (PBCs) are a consequence of their ability to produce free radicals. The proteins embedded in membranes which control ionic gradients across both plasma and organellar membranes are especially easily damaged when oxidized by PBCs. When rats were treated with PBC (Aroclor 1254; 2 mg/kg daily for 30 days) neural levels of lipid peroxidation products along with concentrations of •OH and H_2_O_2_ were elevated. Conversely, GSH concentrations as well as the activities of a variety of enzymes (Na^+^K^+^ATPase, Ca^2+^ATPase, Mg ^2+^ATPase and acetylcholinesterase) were diminished [255]. Giving melatonin (either 5 or 10 mg/kg daily) in combination with the PCB reversed the effects of PBC. In a follow-up study where Aroclor 1254 was used to induce neuronal damage and suppress CuZnSOD and GPx-4 mRNA expression, melatonin again relieved the effects of the PCB [256]. In this study, the benefits of melatonin against PCB neurotoxicity were seen in the cerebral cortex, the hippocampus and cerebellum. In both studies, the protective actions of melatonin were attributed to its antioxidative actions.

## KAINIC ACID

Kainic acid is a neurotoxin isolated from sea weed. When injected into animals it mimics the action of the excitatory amino acid neurotransmitter, glutamate [166]. Kainic acid produces acute and subacute epileptiform activity that can last for days. Ultimately, it causes widespread irreversible neuropathological changes that involve both neurons and glia [231]. Kainic acid binds to and activates a subtype of ionotropic glutamate receptor. In addition to inducing brain lesions directly, probably *via *processes involving free radicals, kainic acid also provokes the discharge of potentially neurotoxic quantities of glutamate from nerve endings [54].

Given that free radicals are likely involved in the neurodestructive processes of kainic acid, soon after melatonin was discovered as a free radical scavenger, it was tested for its antiexcitatory efficacy. In the initial study, when cerebellar granule neurons were incubated with kainic acid the resultant destruction of the neurons was obvious; the toxicity, however, was prevented when melatonin had been added to the incubation medium [83]. In this study the authors documented that melatonin did not directly inhibit the action of kainic acid on glutamate receptors.

*In vivo* as well, melatonin reduced the ability of kainic acid to kill neurons in both the cerebellum and in the hippocampus [85, 238]. Moreover, melatonin reduced the associated neural lipid peroxidation as well as preventing the death of the majority of the rats that received kainic acid [84, 85, 238]. Not only pharmacological amounts of melatonin, but likewise pinealectomy, which only depletes physiological concentrations, enhances the severity of neural damage induced by kainate [145]. The authors of these reports were convinced that the beneficial effects of melatonin against neurotoxicity of kainic acid were due to melatonin’s ability to readily enter the brain and scavenge free radicals and stimulate antioxidative enzyme activities in the affected neurons. The figures in the Tan *et al.* [238] report illustrate the marked protective action that melatonin has on hippocampal pyramidal neurons that are normally damaged by kainic acid. The ability of melatonin to shield these neurons from free radical damage is of particular interest since pyramidal neurons are also lost, *via *mechanisms involving oxidative and nitrosative stress, in a variety of neurodegenerative diseases.

In cerebellar granule neurons, Dabbeni-Sala *et al.* [57] found that kainic acid increased mitochondrial free radical generation (identified with 2’, 7’-dichlorofluorescein) and impaired the function of complex II of the respiratory chain. Moreover, kainate promoted the activity of nitric oxide synthase thereby elevating the production of NO• and ultimately of ONOO^-^. In this series of *in vitro* studies, melatonin proved effective in combating both the oxidative and nitrosative stress associated with the exposure of cerebellar granule neurons to kainic acid.

Follow-up studies have consistently confirmed the efficacy of melatonin in reducing free radical-mediated toxicity due to kainate administration to animals. Confirmation of mitochondrial level of action of melatonin during excitotoxicity was provided by the observations that melatonin also prevented mitochondrial DNA damage [156, 267] while also reducing seizures that often accompany kainic acid administration [267]. Neuronal death that occurs in these situations is presumably the result of apoptosis.

Chuang and co-workers [49] provided direct evidence that in fact apoptosis was the process that leads to cellular implosion after kainate is given. Three days following the intrastriatal injection of kainic acid, these workers noted the dramatic cytotoxic action of the drug along with elevated Bcl-2 immunoreactivity in TUNEL-positive cells. Although detectable, less severe damage was seen in the ipsilateral substantia nigra. Melatonin reduced Bcl-2 expression and preserved neuronal viability (reduced the number of TUNEL-positive neurons). Similar effects of melatonin on Bcl-2 expression in kainic acid-treated rat hippocampal neurons were reported by Yalcin and colleagues [265]. In addition to reducing hippocampal neuronal death due to its direct scavenging activity, melatonin may be protective since it stimulates the antioxidative enzymes, SOD and catalase, in these cells as well [8].

Chung and Han [50] surmised that the toxicity of kainic acid in relation to selective neuronal loss in the hippocampus may involve activation of microglia. Thus, besides enhancing the release of excessive amounts of the excitatory amino acid neurotransmitter, glutamate, which leads to damage of the post synaptic neuron, kainic acid may also activate microglial cells and the production of ROS which would further exaggerate the neuronal damage. Also, since free radicals were presumed to be involved and given the documented antioxidant properties of melatonin, they also examined the ability of this indoleamine to abrogate radical damage, neuron death and microglial activation. 

When rats were injected with a single 10 mg/kg kainate, 72 hours later they exhibited elevated lipid peroxidation and DNA damage, an increased number of TUNEL-positive neurons, reduced cell viability and elevated numbers of microglia (identified by means of isolectin-B4 histochemistry) and astroglial responses (identified by glial fibrillary acidic protein immunocytochemistry); these changes were most prominent in the pyramidal cells of the hippocampus and in the surrounding neuropil. An accumulative dose of 10 mg/kg melatonin (given as 2.5 mg/kg X 4) to kainic acid-treated rats significantly lowered the resulting pyramidal cell death, lipid and DNA damage, microglial activation and to a lesser degree astrogliosis. TUNEL-positive neurons were especially prominent in the CA1 and CA3 pyramidal neurons after kainate.

The authors [50] feel that melatonin’s ability to protect against the toxicity of kainic acid includes its direct effects within neurons as well as reducing the reactive gliosis which is believed to generate free radicals which also presumably contributed to neuronal damage. Additionally, although not specifically investigated in this study, they feel the anti-inflammatory effects of melatonin may have contributed to its neuroprotective efficacy [39, 209]. 

The results of a subsequent publication also implied that glial elements may be involved in kainic acid-mediated damage in the hippocampus and melatonin’s protective actions may likewise involve glial cells [126]. As with the earlier publication, kainate, injected directly into the cerebroventricle, was found to activate both astroglial cells as indicated by the upregulation of GNDF (glial cell line-derived neurotrophic factor) and microglial elements (evaluated with microglial markers, OX-2); these responses were significantly attenuated when melatonin was also administered. 

In addition to examining glial responses associated with kainate administration, Lee and colleagues [126] also evaluated pyramidal cell death (TUNEL assay), total and phosphorylated Akt and iNOS expression. Melatonin reduced CA3 neuron cell death, upregulated Akt and downregulated iNOS, all of which contributed to preservation of hippocampal pyramidal neurons. This beautifully written paper should be consulted for the purpose of visualizing the preserved CA3 neurons, the activation of the glial elements and the upregulation of Akt by melatonin.

As illustrated in studies summarized in this section, melatonin is highly effective in overcoming the toxicity of kainic acid at the level of the hippocampus. The findings have clear implications for several types of seizures which culminate in the massive discharge of glutamate from mossy fibers onto hippocampal pyramidal neurons often resulting in the death of these cells. The studies summarized here indicate that melatonin, due to its ability to attenuate kainic acid (a glutamate analog) toxicity as well as its efficacy in inhibiting seizures themselves [154, 158, 174, 229], may be useful as a treatment for epilepsy. The evidence to date strongly supports this conclusion.

## DOMOIC ACID

Domoic acid is a kainate receptor which has potent neurotoxic activity. When intravenously administered into rats, it induced neural degeneration, glial activation and elevated iNOS activity in the hippocampus [10]. As with kainate, pyramidal neurons in the CA1 and CA3 subfields as well as those of the dentate hilus were severely damaged (based on Nissl staining). Immunoreactive astrogliosis was identified using GFAP (glial fibrillary acid protein) while OX-2 delineated a large number of microglial cells. Domoic acid-mediated neuronal death, reactive gliosis and iNOS protein expression were all reduced to control levels when domoic acid-treated rats also received melatonin.

One additional study also investigated the ability of melatonin to abrogate the death of cerebellar granule cells following domoic acid administration to mice [78]. The kainic acid analog caused elevated levels of intracellular and mitochondrial calcium levels, increased mitochondrial oxidative stress, changes in mitochondrial membrane potential, cytochrome c release, caspase 3 activation and degradation of poly (ADP-ribose) polymerase which culminated in granule cell apoptosis. Domoic acid was incapable of inducing these changes in mice co-treated with melatonin. Since the toxicity of domoic acid likely involves exaggerated free radical generation resulting in elevated oxidative and nitrosative stress [42], melatonin’s function as an antioxidant is presumed to be involved in the mechanisms by which it reduced the toxicity of domoic acid.

## CYANIDE

A cyanide is a compound that contains a triple bond between a carbon atom and a nitrogen atom. Cyanides that release the cyanide ion are highly toxic. Cyanide toxicity stems from the ability of the ion to inhibit cytochrome c oxidase in the mitochondrial respiratory chain.

The subcutaneous injection of potassium cyanide (6, 8, or 9 mg/kg) dose-dependently induced severe tonic seizures in mice and elevated levels of products of lipid peroxidation in the brain of these animals; this latter observation suggests that the interaction of cyanide with oxidative phosphorylation generates increased numbers of free radicals, likely *via *elevating electron leakage from the respiratory complexes. Yamamoto and Tang [266, 268, 269] found that not only did melatonin reduce cyanide-induced lipid breakdown in the brain but it also limited the number of seizures the mice experienced and reduced the death rate of animals that followed their exposure to the lethal agent. In a combination of *in vitro* and *in vivo* studies, this group also found that melatonin limited cyanide-mediated damage to brain mitochondrial DNA [267]. Treating mice with potassium cyanide was also found to induce neuronal death in the substantia nigra of mice with this response likewise being attenuated by giving melatonin [47].

## PERSPECTIVES AND CONCLUSIONS

Melatonin is an uncommonly functionally diverse molecule and a highly effective direct free radical scavenger and indirect antioxidant [237, 239]. Additionally, it readily crosses the blood-brain barrier and permeates into every cell, i.e., neurons and glia. These observations, coupled with its exceptionally high levels in the third ventricular cerebrospinal fluid [130, 227, 251], are consistent with the likelihood of this endogenously-produced indoleamine is protective of the brain against the onslaught of radicals that are sufficiently reactive to mutilate essential molecules in both neurons and glia. Such protection is critical given the high utilization of molecular oxygen by cells in the central nervous system. 

This review summarizes some of the agents that normally inflict damage at the neural level and reviews the studies documenting the ability of melatonin to shield the brain from oxidative and nitrosative damage. Almost uniformly, although there are exceptions as mentioned herein, melatonin demonstrated its efficacy in safeguarding neurons and glia from the persistent molecular disfiguring that would otherwise occur. The problem with loss of neurons is that, again with few exceptions [77], once these cells undergo either apoptosis or necrosis, they are not replaced. Interestingly, neuron precursors in the brain in those select areas where cellular proliferation does occur, are also stimulated by melatonin [114, 143, 185, 205, 230].

One deficiency with the majority of studies summarized in this review is the absence of tests involving the potential involvement of either membrane receptors and nuclear or cytosolic binding sites in the observed protective actions of melatonin in the central nervous system [202]. Whereas the direct free radical scavenging functions of melatonin require no receptor [88, 236], those related to the stimulation of antioxidative enzymes or enhanced glutathione synthesis may involve one or more of the receptors mentioned above [250]. Moreover, there may be yet unidentified functions of melatonin and its metabolites that contribute to the ability of these molecules to render ROS/RNS inept in mediating molecular damage that involve the receptors or binding sites that have been identified.

Another issue that has not been resolved is whether endogenous, physiological concentrations of melatonin are sufficient to protect tissues, including the brain, from the day-to-day attacks by oxygen and nitrogen-based reactants. In the reports reviewed in this survey, melatonin was usually given in pharmacological concentrations. Of course, this was necessary (and would be for any antioxidant) because of the very large increases in free radical generation resulting from the injection of highly toxic agents. One cannot expect physiological levels of melatonin, or any radical scavenger, to protect against pharmacological levels of free radicals. If, in fact, this could be accomplished by the amounts of scavengers that normally exist within cells, no oxidative damage would ever occur. Since molecular damage accumulates throughout the life time of virtually all organisms, it is obvious that the radical scavengers (and there are many) normally present within cells are inadequate to stave off all radical attacks.

Finally, given that endogenous melatonin levels fall substantially with age, at least based on blood and pineal levels of the indoleamine [193, 198, 212], then it could be assumed that the loss of this potent antioxidant may contribute to the development of diseases that involve free radical damage as well as to aging itself [33, 38, 177]. It also suggests that exposure to the toxins mentioned in this report would be more serious in older individuals. Whereas this has yet to be experimentally considered, it is something that should be examined.
